# Applying Deep Learning Techniques to Estimate Patterns of Musical Gesture

**DOI:** 10.3389/fpsyg.2020.575971

**Published:** 2021-01-05

**Authors:** David Dalmazzo, George Waddell, Rafael Ramírez

**Affiliations:** ^1^Music Technology Group, Department of Information and Communication Technologies, Universitat Pompeu Fabra, Barcelona, Spain; ^2^Centre for Performance Science, Royal College of Music, London, United Kingdom; ^3^Faculty of Medicine, Imperial College London, London, United Kingdom

**Keywords:** gesture recognition, bow-strokes, music interaction, CNN, LSTM, music education, ConvLSTM, CNN_LSTM

## Abstract

Repetitive practice is one of the most important factors in improving the performance of motor skills. This paper focuses on the analysis and classification of forearm gestures in the context of violin playing. We recorded five experts and three students performing eight traditional classical violin bow-strokes: *martelé, staccato, detaché, ricochet, legato, trémolo, collé*, and *col legno*. To record inertial motion information, we utilized the *Myo* sensor, which reports a multidimensional time-series signal. We synchronized inertial motion recordings with audio data to extract the spatiotemporal dynamics of each gesture. Applying state-of-the-art deep neural networks, we implemented and compared different architectures where convolutional neural networks (CNN) models demonstrated recognition rates of 97.147%, 3DMultiHeaded_CNN models showed rates of 98.553%, and rates of 99.234% were demonstrated by CNN_LSTM models. The collected data (quaternion of the bowing arm of a violinist) contained sufficient information to distinguish the bowing techniques studied, and deep learning methods were capable of learning the movement patterns that distinguish these techniques. Each of the learning algorithms investigated (CNN, 3DMultiHeaded_CNN, and CNN_LSTM) produced high classification accuracies which supported the feasibility of training classifiers. The resulting classifiers may provide the foundation of a digital assistant to enhance musicians' time spent practicing alone, providing real-time feedback on the accuracy and consistency of their musical gestures in performance.

## 1. Introduction

The purpose of this study was to investigate how state-of-the-art machine learning techniques can be applied to sensor-based, multimodal recordings to complement and enhance the learning of musical instruments. Using violin performance as a test-case, we aimed to provide real-time feedback to musicians regarding their bowing technique, using expert models as reference. This work is a collaboration between the Music Technology Group (MTG) at the Universitat Pompeu Fabra, the University of Genova, and the Royal College of Music, London.

To determine the quality of a gesture performed by a musician is a challenge that involves many factors, not only in terms of motor variability and motor learning. Musicians, whether amateurs or those that strive for professional careers, must internalize an immense amount of information regarding how to read, create, interpret, analyse, memorize, and perform music. This process is often guided by an expert in a classroom or one-to-one setting; a master-apprentice model in which knowledge is passed from one generation of expert to the next. However, most musicians spend the majority of their time away from the expert in their own practice spaces. Thus, they do not have external, consistent, expert feedback on their performance. Such feedback is particularly important in the cycle of self-regulated learning, where good practice is defined by the planning of deliberate goals and strategies to achieve them, the careful execution of these strategies, and, crucially, monitoring and evaluating their performance to determine whether the goals have been met and whether the strategies used are effective (Hatfield et al., 2017). As musicians become expert, they improve their ability to self-evaluate; until they reach this point, it is the role of the teacher to diagnose and provide feedback on a developing musician's performance skills. The technological approach described in this study does not seek to replace the role of music teachers. Rather, it aims to extend the influence of an expert teacher into the practice space so that musicians can learn more efficiently and avoid developing bad motor habits that must be unlearned in each session with a teacher. Should musicians be able to learn complex motor gestures and techniques more efficiently, it can also free time to spend developing creativity and exploring the vast repertoire available to musicians.

The question also remains as to whether musicians, particularly those studying the centuries-old traditions of the violin and related instruments, will engage with such a system. While technology plays a central role in the creation, production, recording, performance, and dissemination of music, it is conspicuously absent in the domain of a music student and teacher were the instrument, the musical score, a metronome, a tuner, and perhaps an audio- or video-recording device may be the only technologies present, and all of which might be present on a single mobile device. However, Waddell and Williamon (2019) found in an international survey of student and professional classical musicians that there was an openness to new technologies to enhance musical learning so long as they were both easy to use and useful. They also found that, while technology was frequently used to monitor and develop aspects of rhythm and intonation (i.e., the metronome and the tuner) there was a gap in use of technologies to address the physical aspects of music-making. This is a gap that the present technology, with further development, might fill.

To address this challenge, we focus on the implementation of artificial intelligence (AI) and machine learning (ML) techniques that have been applied to human gesture recognition across numerous domains with significant impact on human-robot/computer interaction, human activity recognition (HAR), music generation and interaction, and motor learning. Capturing the temporal dynamics of performance gestures requires spatial-temporal event analysis; thus, deep learning architectures with an emphasis on time-series forecasting and classification are widely used, particularly Long-Short Term Memory models (LSTMs), Gated Recurrent Units (GRUs), or hybrid Convolutional Neural Networks paired with LSTM (CNN-LSTM). In the field of human-computer interaction in music, real-time gesture recognition has been reported utilizing ML models that allow precise temporal gesture estimation with just a few samples of reference (Françoise et al., 2012; Caramiaux and Tanaka, 2013; Caramiaux et al., 2013). Caramiaux et al. (2015) presented an ML model for gestural estimations in real-time without the need to define the beginning of an action based on a Sequential Monte Carlo inference. Françoise et al. (2014) have developed useful ML abstractions within the Max/MSP community, proposing Gaussian Mixture Models (GMM) and Hierarchical Hidden Markov Models (HHMM) as temporal likelihood sequential descriptors by defining states of probabilities to fulfill a specific gesture.

In a previews publication (Dalmazzo and Ramirez, 2019b) we have presented an implementation of Hidden Markov Model architecture which provided a foundation to the current study by recognizing bow-gesture patterns in a professional violinist. In this study we described gestural probability fulfillment states through trajectories, providing an accuracy per gesture of (a) 96.3%, (b) 95%, (c) 99.9%, (d) 95.1%, (e) 95.5%, (f) 72.5%, and (g) 88.2% for *detaché, martelé, spiccato, ricochet, sautille, staccato, and bariologe*, respectively. In the next publication (Dalmazzo and Ramirez, 2019a), we described a block of HMM chain to recognize bow-stroke gestures applying a parallel observation of ten different gestures from an expert dataset; however, this approach has some limitations as the gesture is described by limited reference samples. From this perspective, in this current study, we propose a more generic technique to compose a system that can learn the spatiotemporal features that constitute a bow-stroke gesture applying deep neural network algorithms.

## 2. Related Work

### 2.1. Human Activity Recognition

Human Activity Recognition (HAR) is applied in both theoretical research and actual industrial applications. Research has been undertaken in health human activity monitoring (Wearables, 2017), smart homes (Ahmed and Kim, 2016), and human-computer interaction (Xu, 2017). HAR academic practices promote the development of open public datasets (Anguita et al., 2013), fostering the implementation of Deep Learning architectures. Researchers commonly use the “Opportunity” benchmark dataset (Anguita et al., 2013) as it contains IMU (intertial measurement unit) recordings from home activities labeled with actions, such as opening devices or objects (door, fridge, dishwasher, drawer, etc.), cleaning a table, drinking from a cup, flipping a switch, etc. Ordóñez and Roggen (2016) proposed an accurate model called DeepConvLSTM to predict seventeen sporadic arm gestures recorded from multimodal wearable IMUs comprising a gyroscope and accelerometers. Patterns were recorded from four subjects where daily activity was categorized and uploaded to the “Opportunity” dataset. Wang et al. (2017) implemented a three-dimensional convolutional neural network (3DCNN) coupled with LSTM to recognize human activity patterns analysing video sequences. Activities were defined in the datasets with labels, such as bowling, drumming, swimming, push-ups, swing, among many others. Guan and Plötz (2017) applied an LSTM architecture fed with IMU data taken from the same “Opportunity” activity recognition dataset. Zhao et al. (2018) proposed a residual bidirectional LSTM (Res-Bidir-LSTM) to tackle the similar problem of recognizing standard human activity, such as walking in a straight line, walking upstairs or downstairs, sitting, standing, laying down, and standing still with 93.6% accuracy. Zebin et al. (2018) presented an LSTM model adding a batch normalization (+dropout 0.2) to increase the recognition accuracy to 92% for six standard daily-life home activities. UK Biobank publishes the dataset (Doherty et al., 2017). Kuppusamy and Harika (2019) proposed a supervised learning model based on LSTM-RNN with an attentional network to recognize patterns from video-recorded sport actions. Wang et al. (2017) introduced a model called scLSTM, which provides a method to generate salience-aware videos to apply 3DCNN-LSTM. The model for video activity recognition consists of 51 action categories, which together contain ~7,000 labeled clips extracted from a variety of sources [HMDB-51 (Kuehne et al., 2011)]s. This research field is an excellent source of DL models to apply to human-computer interaction in the musical context.

### 2.2. Dance Generators

Another field of research involving coder-decoder ML model translators and musical inputs are dance choreography generators. This is an example of how different artistic disciplines can be extrapolated harnessing DL models with similar time-sequence analysis principles. Françoise et al. (2017) developed the GrooveNet framework. It generates dance movements from a specific pre-trained audio reference in real-time, with models based on Factored Conditional Restricted Boltzmann Machines (FCRBMs) and Recurrent Neural Networks (RNNs). Tang et al. (2018) implemented an LSTM-autoencoder model to define a mapping between acoustic and motion features. Jia (2019) presented an automatic music choreography generator implementing a 3-layer LSTM which learns the relationships between quaternion motion data from dance recordings coupled with musical features. Yalta et al. (2019) developed an optimization technique for weakly-supervised deep recurrent neural networks for dance generation. Their model is based on two blocks of LSTMs, where one has the role of reducing the music input sequence (encoder), and the other is for the motion output sequence (decoder). Sun et al. (2020) proposed a Generated Adversarial Network-based cross-modal association framework, which correlates dance motion and music modalities together. The model generates dance sequences from a musical input.

### 2.3. Gestures and Sensors

Inertial measurement unit (IMU) devices are composed of a kit of sensors (e.g., accelerometers, gyroscopes, magnetometers, etc.) and transmit inertial data through Wi-Fi or Bluetooth wireless connections at 200 Hz. IMUs and IR optical sensors such as LeapMotion are the most common tools to capture gestural events for different subjects. Ordóñez and Roggen (2016) have implemented a 3DCNN-LSTM deep neural network to recognize seven gestures (five for finger tapping, one palm tapping, and one pointing) as a model for interactive music applications utilizing the LeapMotion device. Zhang et al. (2017) proposed an architecture based on 3DCNN->LSTMs fully connected (FC) to a 2DCNN pre-output layer and projected to an FC/Softmax final output descriptor. In this study, the analysis was based on color videos divided into 249 labeled gestures performed by 21 participants. Drumond et al. (2018) published a study wherein five action movements were recorded utilizing the *Myo* sensor in a game environment for interaction purposes. The proposed LSTM model had an accuracy of 96%. Seok et al. (2018) presented a reinforcement learning model with the architecture of an LSTM layer with two consecutive Dense FC layers to estimate hand gestures, capturing data from the *Myo* armband. Zhang and Li (2019) confirmed that the CNN-LSTM architecture is suitable for analyzing sequential data gathered from the *Myo*'s electromyogram (EMG) sensors with an accuracy of 98%. Hasson (2019) applied a CNN-LSTM technique to recognize hand gestures labeled as rest, wave in, wave out, spread, fist, index pointing, “1 + 2,” “1 + 3,” “1 + 4,” and scissors, implementing *Myo*'s EMG data. Chen et al. (2020) have also focused on the electromyogram signals from the *Myo* sensor. They proposed CWT+EMGNet, which consists of four convolutional layers, plus a max-pooling layer without a fully connected layer in the output. The gestures are part of the Myo Dataset (Côté-Allard et al., 2019) and NinaPro DB5 (Pizzolato et al., 2017). Guo and Sung (2020) captured human motion utilizing the HTC-VIVE virtual reality device in synchronization with the *Myo* armband. They harnessed the Bi-LSTM and two-layer LSTM architecture to recognize 15 different motor actions using the arms in a 3D video-game context. Gestures are labeled as “exploring the cave,” “running away,” “through the tunnel,” “through the waterfall,” “attacking,” “fighting,” and “capturing equipment,” among others.

### 2.4. Music Gestures and RNN

Hantrakul and Kondak (2018) implemented an LSTM architecture composed of four layers [LSTM(64), LSTM(32), FC(16), and FC(3)] to recognize and predict different hand gesture drawings over a Roli Lightpad Block. Hand gestures are used as an interactive new layer over electronic musical real-time manipulation. The authors have released the code where communication between Ableton live, Wekinator, and Roli lightpad is proposed. Erdem et al. (2020) presented an LSTM-based model to add a new layer of interaction in electric guitar interpretation, by training the system with three specific sound manipulations defined as impulsive, sustain, and iterative. To do so, the authors utilized the *Myo* sensor as an interactive input, reading the electromyogram signals of the performer's forearm, to trigger the sound manipulations. Pati et al. (2018) proposed a hybrid model based on Mel spectrogram analysis from audio recordings of traditional music performance to pass the multidimensional data stream into a convolutional 1D layer projected to a recurrent neural network. The model receives the name of M-CRNN. The main goal of the authors is to propose an RNN model to provide music performance assessment of wind instruments in Western classical music contexts.

## 3. Materials and Methods

### 3.1. Musical Materials

**Figure 1:**
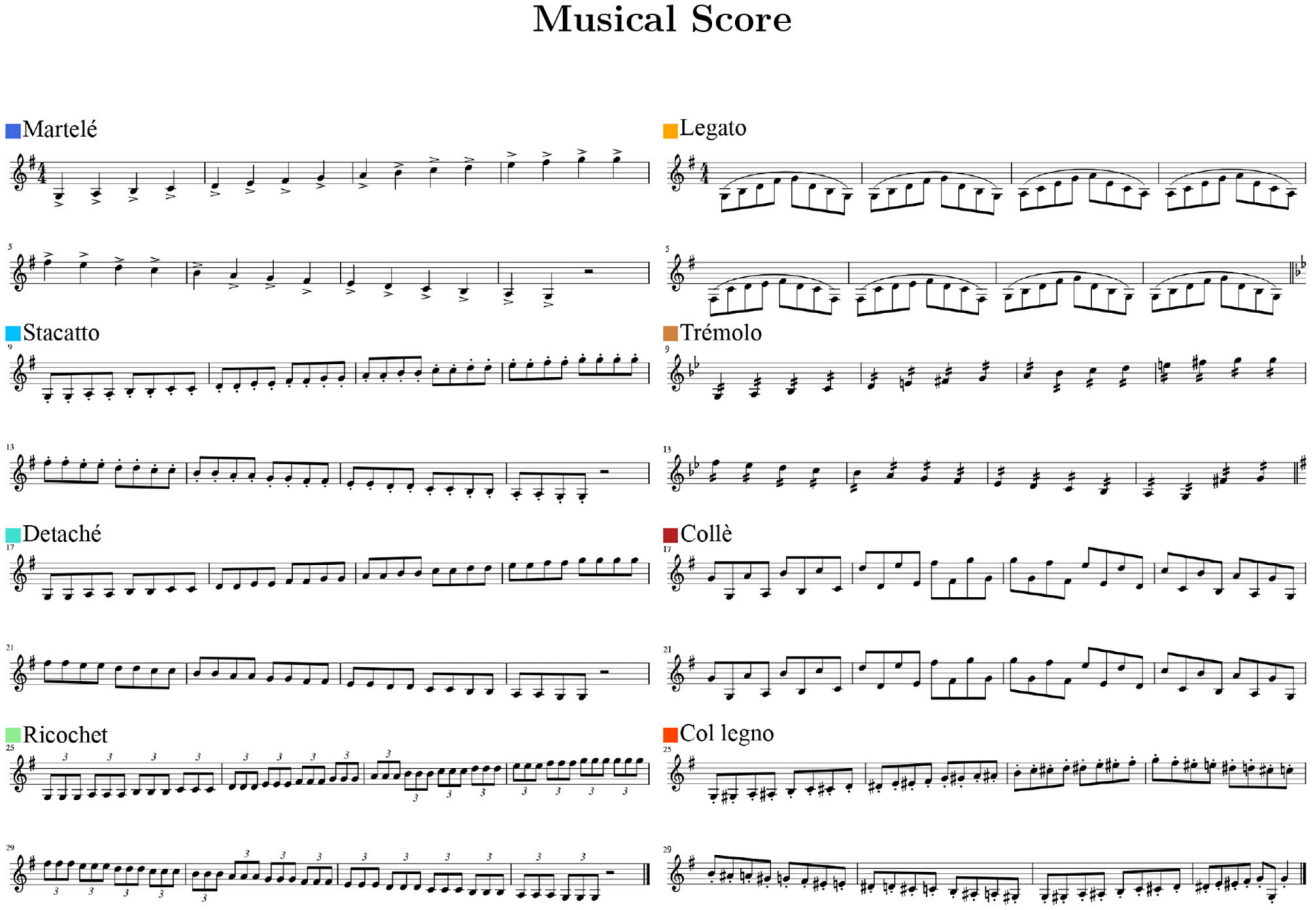
Musical excerpts performed for each of the eight violin bowing gestures.

Eight bow-strokes were recorded by musicians following a musical score (see Figure 1) with a fixed metronome tempo of quarter-notes at 80 bpm. All gestures were established in G, primarily in the major mode except for *tremolo* (G minor) and *col legno* (chromatic). On the violin, two octaves from G3 to G5 covers the whole neck and also all four strings. Eight musicians participated in the recording sessions, with expert models constructed using the data from five violinists; the other three participants were reserved as test cases. The recordings are part of a collaboration with the Royal College of Music in London.

The eight bow-strokes the violinists were instructed to perform comprised:

- ***Martelé:*** meaning *hammered*; an extension of *détaché* with a more distinctive attack, caused by a faster and slightly stronger initial movement to emphasize the starting point with an accent followed by a smooth release and silence between notes.
- ***Staccato:*** a shorter, sharper version of *martelé*. It features a clean attack generated by controlled pressure over the string with an accentuated release in the direction of the bow-stroke. It is controlled by a slight rotation of the forearm where pronation attacks the sound and supination releases it. It is generated from the wrist by an up-and-down motion attack, or a pinched gesture with the index finger and the thumb.
- ***Detaché:*** meaning *separated*; a stable sound produced in each bowing direction. The sound has to be kept dynamically stable, moving smoothly from one note to the next. The weight over the violin strings is kept even for each note performed. It is perhaps the most common bowing technique in the violin repertoire.
- ***Ricochet:*** a controlled bouncing effect that produces a rhythmic pattern, usually comprising two to six rapidly repeated notes. It is played in a single down-bowed stroke starting with a *staccato* attack, while movement of the wrist is used to control the weight of the bow against the violin's string.
- ***Legato:*** created by sustaining the bow though multiple notes, establishing fluency without pause in the sound. The musician avoids excessive emphasis, accents, or attacks. For the musical excerpt, consecutive arpeggios composed of four notes each were recorded.
- ***Tr*é*molo:*** a stroke commonly found in orchestral repertoire where the bow moves back and forth very rapidly, often without any defined meter. For this study, a semiquaver *trémolo* was established as a constraint.
- ***Collé:*** meaning *stuck* or *glued*; created by a heavily weighted bow resting on the string followed by a subtle release. It produces a short attack with a rough sound effect.
- ***Col legno:*** meaning *with the wood*; caused by the percussive strike of the string with the wooden shaft of the bow.

### 3.2. Data Acquisition and Synchronization

- ***Myo***, an IMU device developed by Thalmic Labs for gestural-control human-computer interaction purposes, was used for data acquisition. The IMU bracelet weighs 93 grams with an adjustable diameter of 12.5–38.4 cm; none of the eight participants reported that the device caused any significant impediment their optimal performance. The hardware includes eight medical-grade stainless steel EMG sensors which report raw electrical muscle activity in a voltage range of 0–2 mV expressed in oscillations of −1 to 1 (Hassan et al., 2019). Two battery cells are embedded with a capacity of 260 mA/hr and an operating voltage range of 1.7 to 3.3 V. A three-axis gyroscope records angular velocity in degrees of change in radians per second, and a three-axis accelerometer as an estimation of −8 to 8 g (1 g = 9.81 *m*/*s*^2^). A three-axis magnetometer produces a quaternion defined as q = a + bi + cj + dk (where a, b, c, and d are real numbers and i, j, and k represent the imaginary-number pointing vector); this records rotation of the *Myo* in space. It houses an ARM Cortex M4 processor, and it can provide short, medium, and long haptic feedback vibration. Its communication with the computer is based on Bluetooth with an included adapter, giving a sampling rate of 200 Hz (hop-time of 5 ms). Two *Myos* were placed on both forearms of the participants to record right arm bowing motion and left hand EMGs of movements on the violin neck and strings.
- ***Max/MSP*** is a visual programming language platform commonly used in electronic music and interactive media development and creation, suitable for quick prototyping. It allows communication with external devices. *Myo* is captured in Max/MSP utilizing the external object https://github.com/JulesFrancoise/myo-for-max.
- ***Synchronization*** To record the gestures and synchronize the *Myo* device with the audio data, we implemented a Max/MSP program which recorded *Myo*'s data at 60 fps and audio data with a master trigger-clock to start and stop both recordings in the same folder. The IMU database was formatted as a CSV file. These files were created taking into account a synchronization format: sample counter, time counter reference in milliseconds, accelerometer (x, y, z), gyroscope (x, y, z), quaternion (w,x,y,z), electromyogram (eight values), and MIDI notes. Those CSV files were recorded in the same time-window range reference of the audio data. We programmed an interface for participants to provide coding information then we formatted the names of the recorded files as an automated counter+user_name+gesture_id+second+minute+hour+day+month+year (using .csv and .wav extensions), where the counter was the iteration of the recording session and the gesture was an identification number and time/date description which stacked all files to avoid overwriting. The master recorder in Max/MSP sent the global timer (ms) reference to the *Myo* recorder, which was reported in the CSV file. To acquire audio, we used an Zoom H5 interface linked to Max, recording WAV files with a sample rate of 44.100 Hz/16 bits. The *Myo* device was operated via a MacBook Pro (13-inch, 2017) with a 2.5 GHz Intel Core i7 processor and a memory of 8 GB 2133 MHz LPDDR3 with a latency of 10 ms.
- ***TensorFlow and Python libraries*** To process the data, reorganize and format the final database, and define the ML models, we used Python “3.6.8,” TensorFlow “2.0.0,” NumPy “1.17.4,” Scikit-learn “0.23.1,” and Pyquaternion “0.9.5.”
- ***Repository*** All of the deep learning models, code, and processed data utilized in this research can be tested and reproduced. To access to the repository please visit the link.^1^ The code and datasets are released under Creative Commons Attribution-NonCommercial-ShareAlike 4.0 (CC BY-NC-SA 4.0) license.^2^

### 3.3. Methods

**Figure 2:**
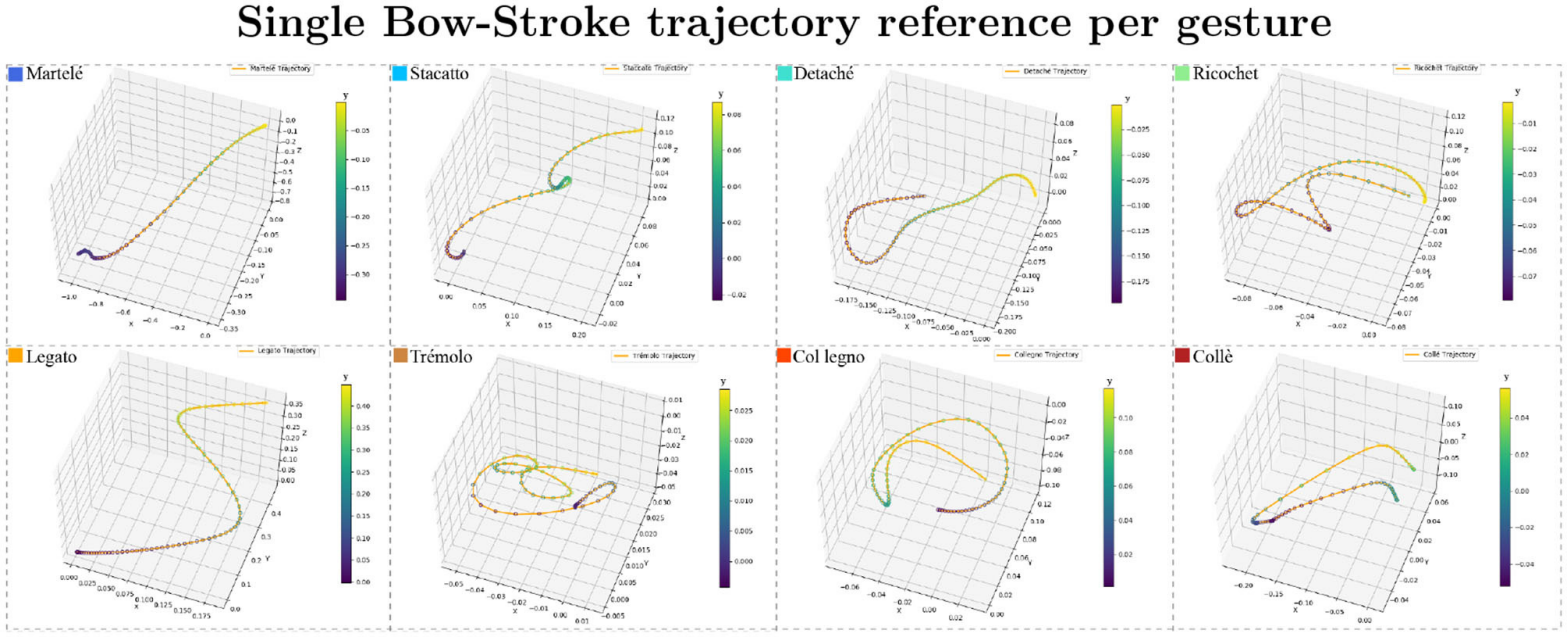
Trajectory samples of all gestures. The bow-stroke examples displayed were chosen randomly from the expert performers. The shapes can be understood as temporal signatures with specific speeds and sounds. The performer's samples are similar in speed and shape but not identical. The color bar is the reference of the depth shown as the “y” axis.

**Figure 3:**
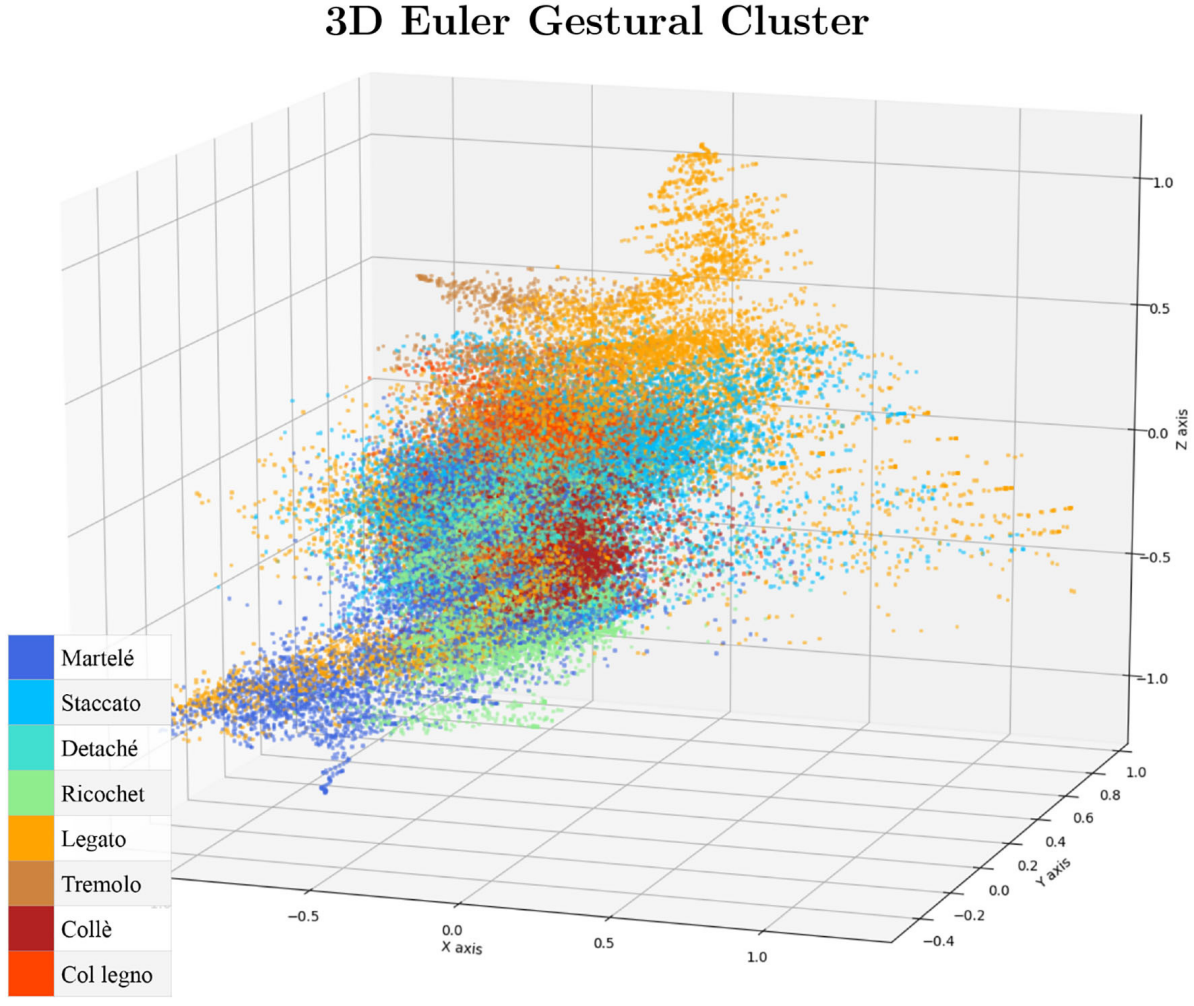
The cluster visualization serves to check if the data distribution is centralized and normalized.

**Figure 4:**
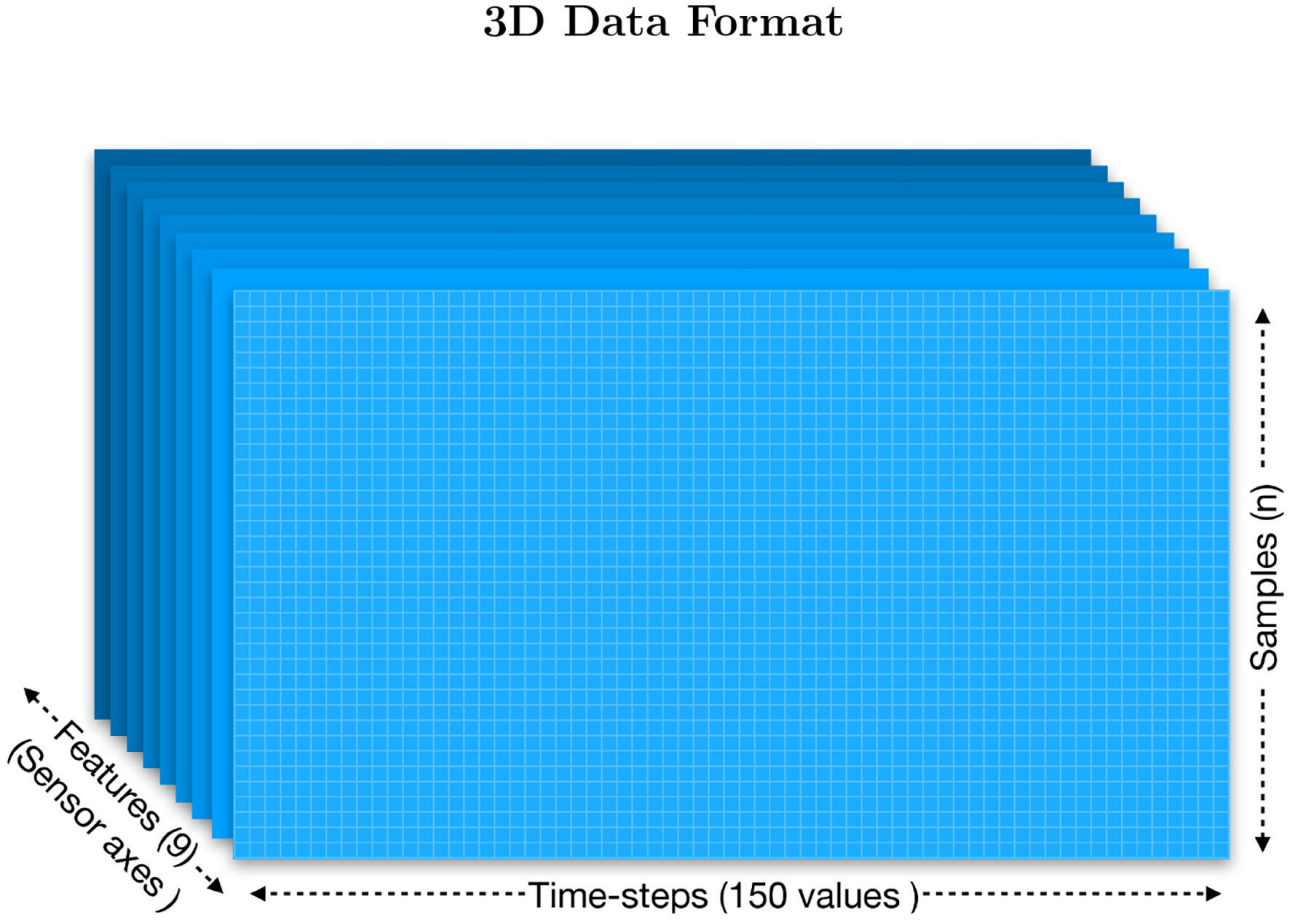
The three-dimensional data is organized in sets of 150 samples which contain two bow-strokes per sample. The x-axis is the observation of those paired bow-strokes, the y-axis is the number of observations defined as “samples,” and the z-axis is the number of features, which in this case is 3 × 3 sensor axes (gyroscope, accelerometer, and Euler-angles). Hence, each of the features is itself a file of time-steps × samples stored in a folder.

**Figure 5:**
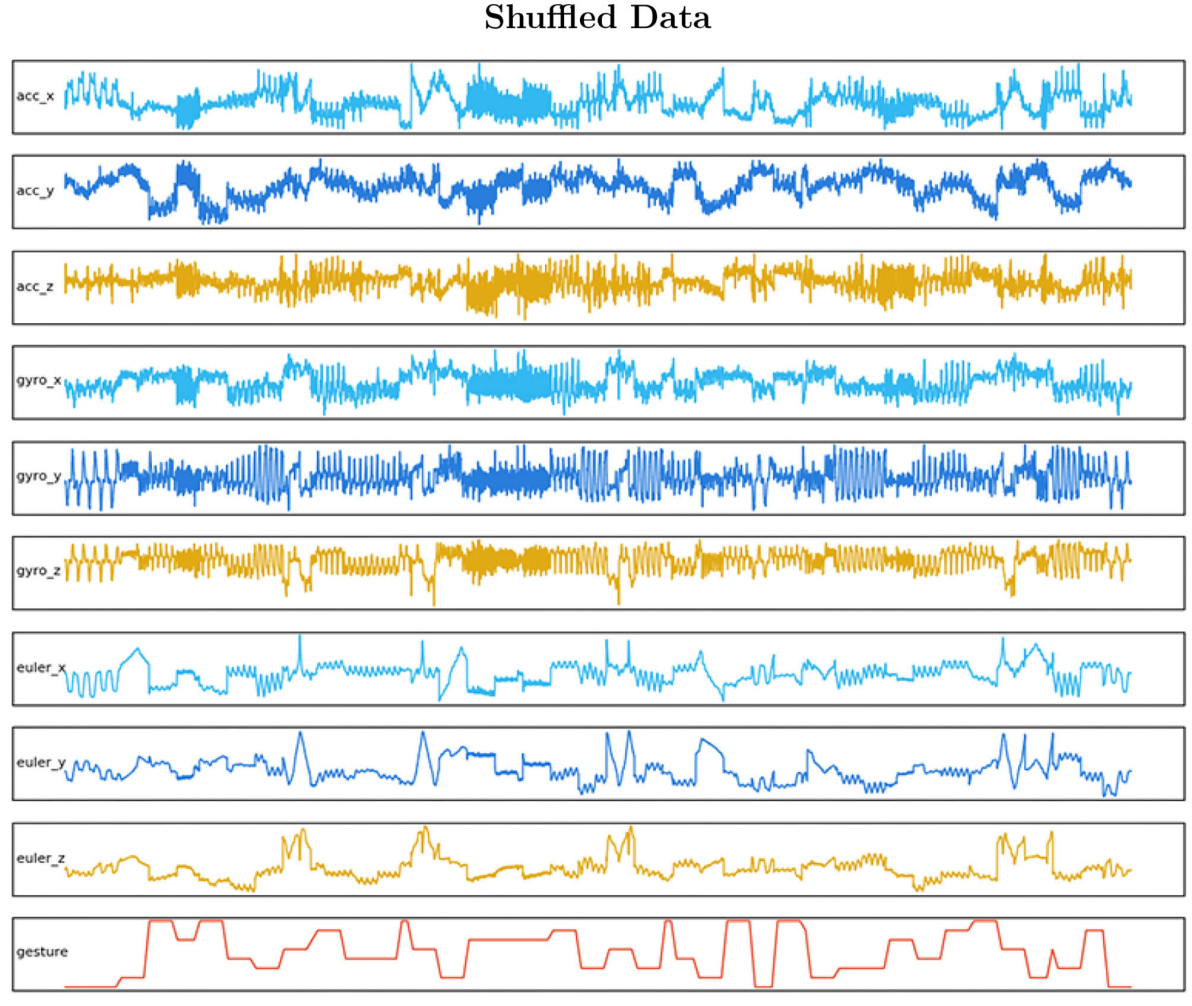
The shuffled data gives an insight into how the features and the labeled datasets have to be reorganized with the same sample order. By shuffling, we ensure that each data observation creates an independent unbiased change on the model, learning all gestures in the same proportion.

- **Data Preparation**: The steps to prepare the data were:
  – translate quaternions to normalized and centralized Euler 3D orientation;
  – organize the data into a three-dimensional format;
  – create a windowed dataset;
  – define the labels;
  – shuffle the packages of windowed data;
  – format the data to supervised learning.
- **Translate quaternions to normalized and centralized Euler 3D orientation**: *Myo*'s orientation is given by a quaternion as x,y,z,w. Its orientation formula is *q* = *a* + *bi* + *cj* + *dk* formed by a real-numbers component expressed in the letters a, b, c, d and imaginary-number components expressed as i, j, k as a pointing vector along the three spatial axes. It can also be expressed as:
  q=cos(θ)+sin(θ)(i+j+k)
  For visualization purposes, the quaternion is reoriented to a defined origin to match the computer screen and the performer forearm angle. If the “orientation” desired is given by *q*(0.93, 0.0, 0.0, −0.36), then the *origin* will be given by the first sample of the performer. The *performerQuaternion* is the gestural data array.
  $$\begin{array}{ll} result=orientation*performerQuaternion*origin.inverse\\ {}* & orientation.inverse \end{array}$$
  Finally, the result is transformed into a normalized 3D vector as Euler angles (yaw/pitch/roll) as is shown in Figure 2. For a cluster visualization of the normalized data see Figure 3. Further information on quaternion transformations can be found on the website of *3Blue1Brown* and Ben Eater.^3^
- **Organize the data into a three-dimensional format**: The *Myo*'s raw data for each subject is stored in a CSV table (2D) with more than 2,800 samples (n) and 22 columns. The file header is: (sample, timer, acc_x, acc_y, acc_z, gyro_x, gyro_y, gyro_z, quat_x, quat_y, quat_z, quat_w, emg_0, emg_1, emg_2, emg_3, emg_4, emg_5, emg_6, emg_7, gesture_id, note). To organize the data, we first discarded the samples that did not belong to a specific bow-stroke. The “note” column was used to identify where the gestures were performed as it provides the reference of the MIDI note given by the musical score being performed. As a result, there is a performing window of 75 samples per gesture, which has 0.375 s of range, and 30 gestures as a good reference of window-observation per each exercise/gesture recorded. We then discarded the EMG features to train using only the inertial motion data. Next, all data from all subjects were recollected to extract independently the sensor-axes, which served as the “features.” They are three sensors (acc, gyro, and Euler) multiplied by three axes, totalling nine independent files, each of them as tables with shape [n,150]. Both architectures, Convolutional Neural Networks (CNN) and the Long-Short Term Memory (LSTM), expect three-dimensional formatted data, which is defined by [**Samples**, **Time-steps**, **Features**]:
  – **Samples**: the sequence of the windowed data.
  – **Time-steps**: one time-step is the definition of a window range as a point of observation of the sequence.
  – **Features**: the number of different observations; in this case, the axis of each sensor.
- **Create a windowed dataset**: The data characteristics comprised fixed windows of 0.375 s, given 150 data points to observe, with 50% overlap as shown in Figure 4. This method is a standard data augmentation technique. The format was then stored in a folder containing nine matrices made of a single sensor axis. All files (acc_x, acc_y, acc_z, gyro_x, gyro_y, gyro_z, euler_x, euler_y, euler_z) were made of samples(n), time-steps(150). For instance, after dividing the data into training and test by 80–20 %, we have an **input_shape** for training data of [940,150,9].
- **Define the labels**: Labels were extracted from the gesture column. A new file was then created that matched the n Samples shape. They provided the reference class for each sample.
- **Shuffle the packages of windowed data**: The data were then shuffled in groups of five consecutive Samples. A defined shuffle array was created as a file, then its values were passed as a pointer reference to both datasets to the [samples, time-steps] array and the labels array. This method will reorganize both matrices in a predefined shuffle. In Figure 5 the final data are visualized.
- **Format the data to supervised learning**: Supervised learning is applied to the LSTM forecasting time-series model. As we are working with nine features which will output eight different gestures with a time-step of 75 data-point observations, we need to work in a Multivariate Time Series format. The sliding window with multiple steps technique is applied with a window range of 75 samples to divide the dataset for training and test into consecutive sequential batches where the test observation has as an output to the next 75 windowed data-points. In other words, we define that the gesture is completed in a range of 75 samples. That range is defined as a batch. Hence, we can compare the next batch execution to the previews one by estimating how accurate the LSTM model expressed the trajectory. That comparison is defined as a supervised learning technique.

### 3.4. Classification Models

**Figure 6:**
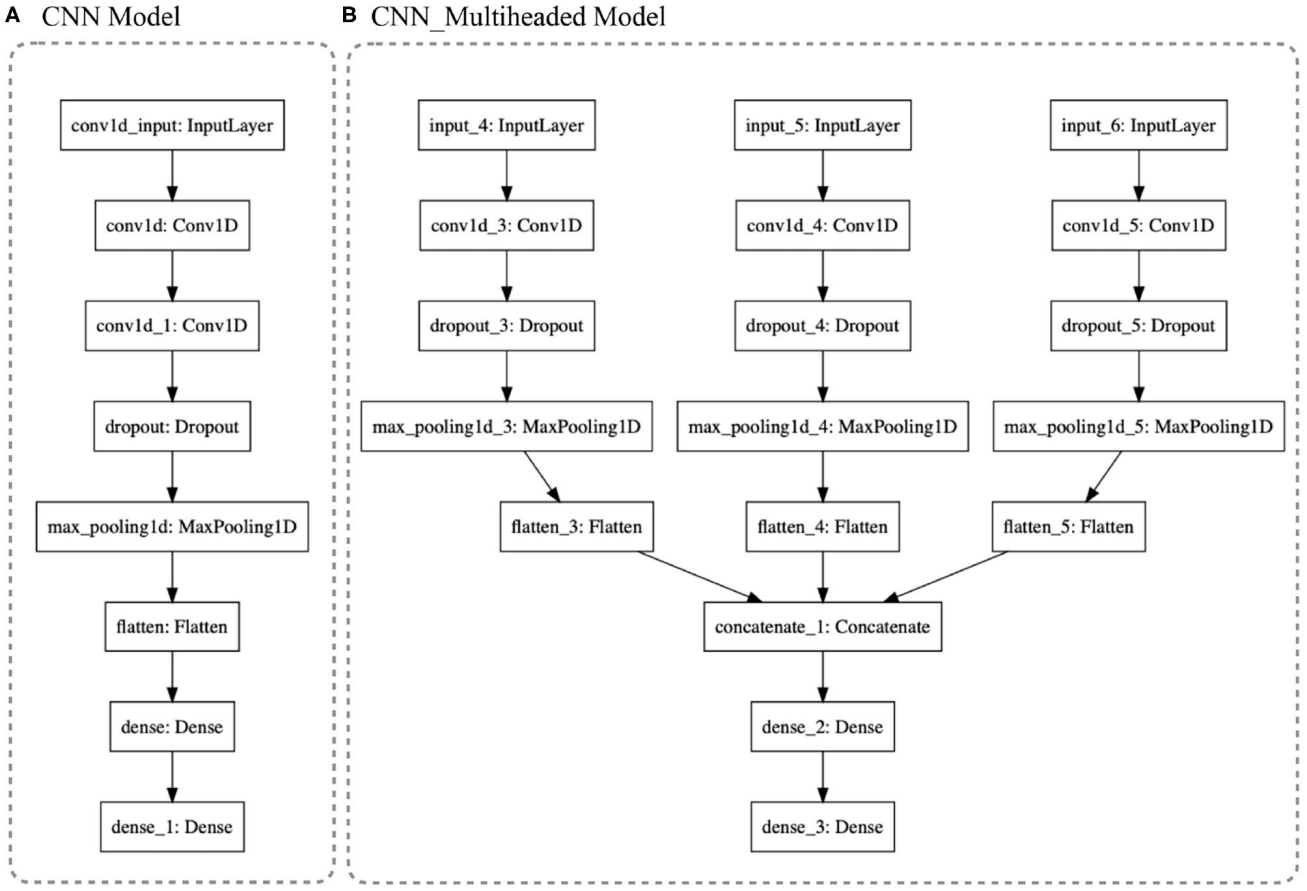
**(A)** CNN architecture: After the filtering layers, with a dropout of 0.5, the first Dense layer is 100 neurons in size projected to eight neurons of output. **(B)** 3D_Multihaded_CNN: Each head is a different resolution of the whole package of data which is concatenated in the layer concatenate_1.

**Figure 7:**
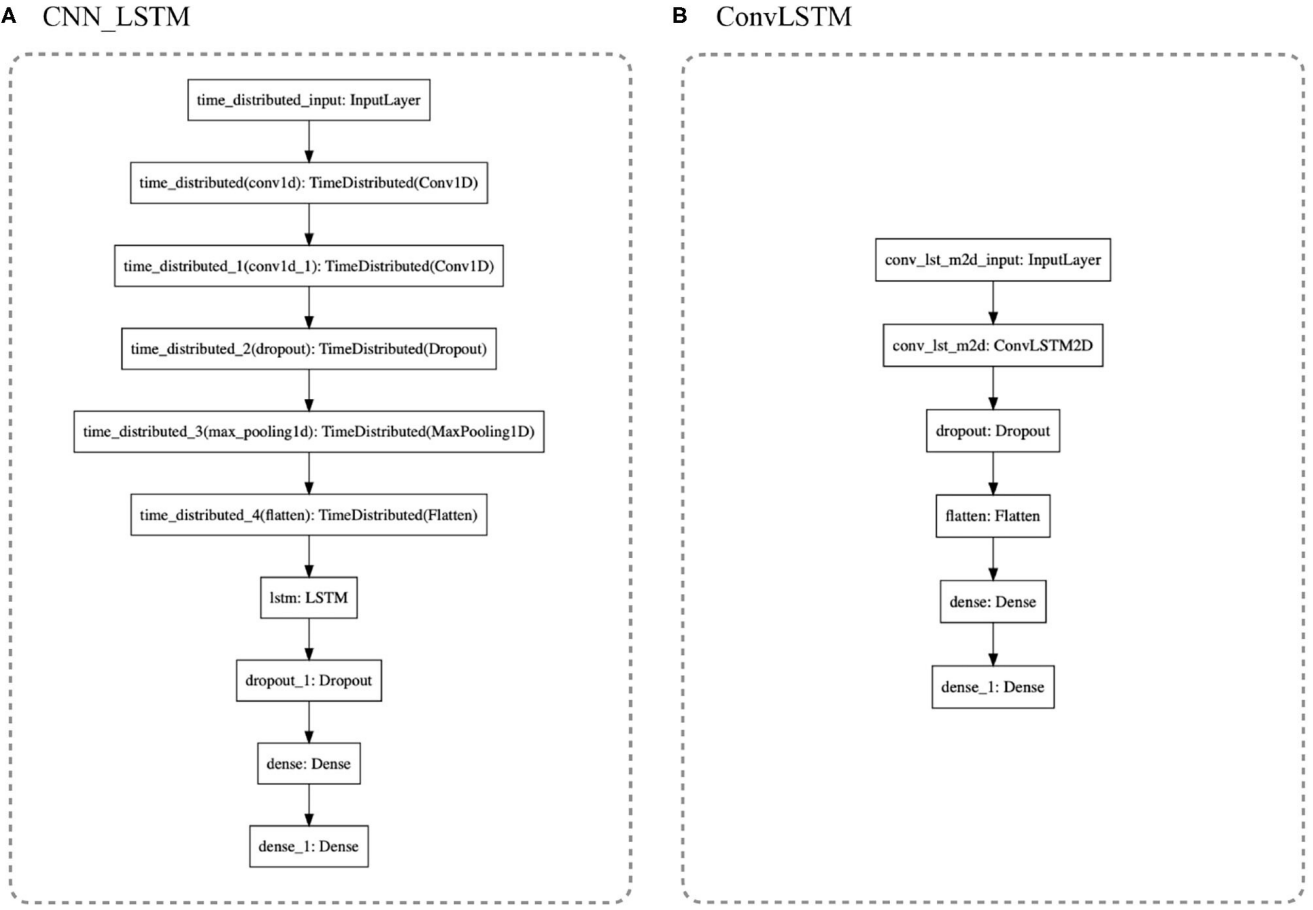
**(A)** CNN_LSTM is a hybrid model with six layers of CNN processing extracting temporal features of the gestures and projecting them to a standard Vanilla LSTM. **(B)** ConLSTM is a recurrent neural network LSTM that handles 3D tensors, receiving in the input gates the matrices processed by its internal CNN.

- **Traditional Machine Learning Models**: To test and compare different approaches to the problem of estimating which gesture is being performed, we implemented the traditional ML models found in the literature. The main idea was to define a background starting point of gestural-estimation accuracy. Based on the default Scikit-learn python library (Pedregosa et al., 2011), non-linear-algorithms were tested: (1) k-Nearest Neighbors, (2) Classification and Regression Tree, (3) Support Vector Machine, and (4) Naive Bayes. For the ensemble-algorithms: (5) Bagging Classifier, (6) Decision Trees, (7) Random Forest, (8) Extra Trees, and (9) Gradient Boosting Machine. We also tested Hierarchical Hidden Markov Models in a previous publication (Dalmazzo and Ramirez, 2019b).
- **CNN Models**: We tested three CNN models: (1) CNN, following the standard architecture applied in Human Activity Recognition (HAR); (2) the same CNN model with data Standardization; and (3) a Multi-headed CNN model. In Figure 6, the first two models used the architecture shown in (a) and the Multi-headed model is given by (b). The standardization is the normalization of the data to have a mean centered at zero and a standard deviation of 1. Taking into account that the motion data was recorded as a variation from an origin with a Gaussian distribution, it is possible to apply the technique. This serves to enhance the formation of the learned features. The models are composed of 1D Convolution layers as they are extracting features from each sensor channel independently, instead of the traditional 3D Convolution where each dimension is the package of red, blue, and green channels of video data streams (see Figure 6). Six different CNN filters were tested for architecture optimal accuracy: filter = [8, 16, 32, 64, 128, 256]. Also, five versions of kernel sizes were tested: kernel = [2, 3, 5, 7, 9]. **The Multi-headed**: CNN model was composed of three different sized kernels. The main idea was to process the data in three different resolutions; however, the interpretations are concatenated by a fully connected layer which projects to the Dense layer that will define the output. See Figure 6.
- **LSTM Models**: Three state-of-the-art models were included: Vanilla Long Short-Term Memory Recurrent Neural Network, One-dimensional Convolutional Neural Network LSTM (CNN_LSTM), and One-dimensional Convolutional LSTM (ConvLSTM). **Vanilla LSTM** is a single hidden layer of LSTM and it reads one time-step of the sequence at a time. The model forms its own representations by passing the time-steps through different cells, maintaining and remembering the features that are relevant through the cycles and forgetting those representations that do not pass a *sigmoid* forget gate. It is capable of learning long-term dependencies of patterns. The standard model is given by:
  (1) $$\begin{array}{l} f_{t}=\sigma_{g}\left(W_{f}x_{t}+U_{f}h_{t-1}+b_{f}\right) \end{array}$$
  (2) $$\begin{array}{l} i_{t}=\sigma_{g}\left(W_{i}x_{t}+U_{i}h_{t-1}+b_{i}\right) \end{array}$$
  (3) $$\begin{array}{l} {\tilde{c}}_{t}=\sigma_{h}\left(W_{c}x_{t}+U_{c}h_{t-1}+b_{c}\right) \end{array}$$
  (4) $$\begin{array}{l} c_{t}=f_{t}◦c_{t-1}+i_{t}◦{\tilde{c}}_{t} \end{array}$$
  (5) $$\begin{array}{l} o_{t}=\sigma_{g}\left(W_{o}x_{t}+U_{o}h_{t-1}+b_{o}\right) \end{array}$$
  (6) $$\begin{array}{l} h_{t}=o_{t}◦\sigma_{h}\left(c_{t}\right) \end{array}$$
  The first cell is a *Forget Gate* layer. It observes *h*_*t*−1_ and *x*_1_, giving as an output a number from 0 to 1 (*c*_*t*−1_). The value is the weight of the forgetting cell; when it is zero, the observation is discarded. The next step is the intersection (*i*_*t*_) called *Input Gate* that decides what information is going to be stored in the next cell. The third gate is called *Cell State*. It updates the old observation ${\tilde{c}}_{t}$ into the new *c*_*t*_ multiplying the Forget Cell, adding it with the old *c*_*t*_ intersection. The next step is to pass the result into a *tanh* function to express the values in a −1 to 1 range. The last gate is the *Output Gate* with the following layer called hidden state (*h*_*t*_). The result will be passed to the input gate in the next cycle. It multiplies the *tanh* output with the *sigmoid* output to decide which information should be carried in the next step to be compared with the new observation input, giving a long-term memory cell.
  **CNN_LSTM**: It is a hybrid model of a convolutional neural network passed to a LSTM's cell (see Table 1). Two layers of 1D Convolution are used to extract temporal-sequence features that will be given to an LSTM layer that will remember the local features extracted from the CNN to keep updating the classification models. For this architecture, the multivariate data is formatted as [samples, sub-sequences, time-steps, features].
  **ConvLSTM**: was developed to read two-dimensional spatial-temporal data. It expects an input-shape of [samples, time-steps, rows, columns, features]. For this study, the rows were translated to number-of-sequence and columns into number-of-steps (the length of the sequence). For further insight into the DNN architecture, see the python code reference (GitHub). The architecture is outlined in Figure 7.

**Table 1:** Model: sequential CNN_LSTM.

**Layer**	**(Type)**	**Output shape**	**Param#**
Conv1D	(TimeDistributed)	(None, None, 48, 32)	896
Conv1D	(TimeDistributed)	(None, None, 46, 32)	3,104
Dropout_1	(TimeDistributed)	(None, None, 46, 32)	0
MaxPooling1D	(TimeDistributed)	(None, None, 23, 32)	0
Flatten	(TimeDistributed)	(None, None, 736)	0
LSTM	(LSTM)	(None, 100)	334,800
Dropout_2	(Dropout)	(None, 100)	0
Dense	(Dense)	(None, 100)	10,100
Dense_1	(Dense)	(None, 8)	808

*Total params: 349,708*.

*Trainable params: 349,708*.

*Non-trainable params: 0*.

## 4. Results

**Figure 8:**
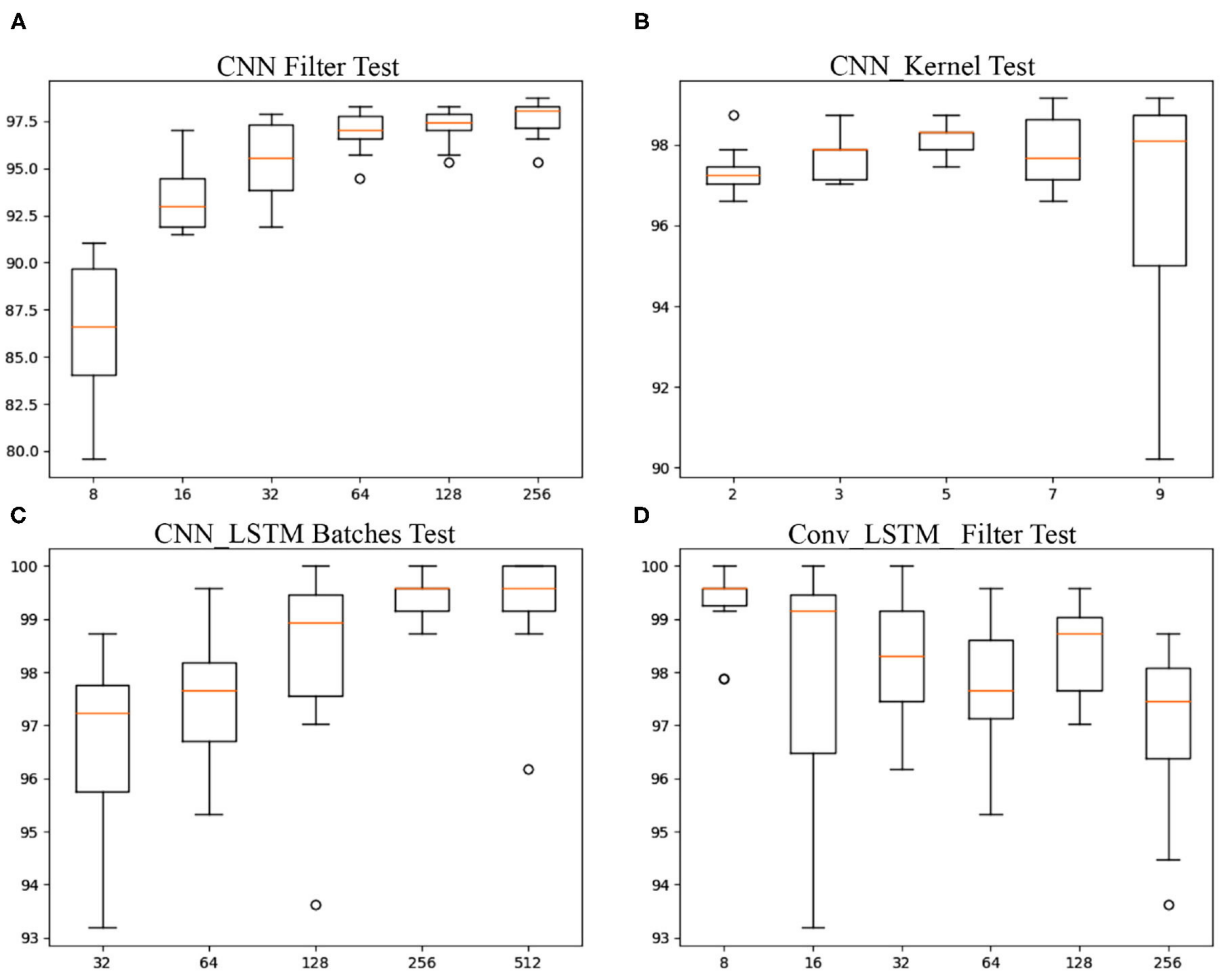
Boxplot of accuracy reports from **(A)** CNN filter configurations of 8, 16, 32, 64, 128, 256; **(B)** CNN kernel configurations of 2, 3, 5, 7, 9; **(C)** CNN_LSTM Batches configurations 32, 64, 128, 256, 512; and **(D)** Conv_LSTM with filters configurations of 8, 16, 32, 64, 128, 256. All models were run 10 times to determine their range of accuracy.

We addressed the challenge of recognizing bow-stroke gestures utilizing data acquired from forearm *Myo* sensor recordings from expert performers. By sending the nine features observations to ML and DNN models, we correctly classified and estimated eight standard violin bow-strokes with the following results:

- **Traditional Machine Learning**:
  After training nine ML classifier models included in the Skit-learn python library, we produced the following accuracy report, organized from highest to lowest: 97.447% with Gaussian Process, 95.319% with Extra Trees, 94.468% with Random Forest, 93.191% applying Bagging classifier, 92.340% with Gradient Boosting, 90.638% with K-Nearest-Neighbors, 86.809% with Support Vector Machine, 71.915% using Decision Tree, and 61.277% with Naive Bayes classifier. The training parameters were defined via testing by-default references from the Skit-learn tutorials. The code can be found in the GitHub repository^4^ (see Table 2).
- **CNN**:
  The CNN architecture reported a percentage of correct classified gestures of 96.979% (sd. ± 0.922). The same CNN architecture with data Standardization, had an accuracy of 97.149% (sd. ± 0.809). We tested different parameter configurations. As an experiment, we ran the model ten times with each of the parameters to estimate a mean and standard deviation of correct classified gestures: The filters we tested defined the size of the first convolutional layer. For the Filter-Parameter (FP) *fp* = 8, the accuracy reported was 86.383% (sd. ± 3.651). *fp* = 16: 93.319% (sd. ± 1.713). *fp* = 32: 95.447% (sd. ± 2.059). *fp* = 64: 96.894% (sd. ± 1.077). *fp* = 128: 97.234% (sd. ± 0.956). *fp*=256: 97.617% (sd. ± 0.993). See Figure 8A. The same approach of running the model ten times was applied to five different Kernel-parameters (KPs) (2,3,5,7,9). The parameters reported a percentage of correct gesture estimations of: *kp* = 2: 97.319% (sd. ± 0.603). *kp* = 3: 97.702% (sd. ± 0.545). *kp* = 5: 98.170% (sd. ± 0.383). *kp* = 7: 97.830% (sd. ± 0.817). *kp* = 9: 96.723% (sd. ± 2.738). See Figure 8B. The Multi-Headed_CNN model with each cnn_head filters defined as 3,5,9, correspondingly, had an improvement of accuracy to 98.553% (sd. ± 0.340).
- **LSTM**: Three LSTM models were tested, first a Vanilla LSTM with a classification and regression accuracy of 86.383% (sd. ± 5.516). The second model was a Conv_LSTM with six different batch-sizes (BZ) (8, 16, 32, 63, 128, 256) having the report of correct gesture estimations of: *bz* = 8: 99.234% (sd. ± 0.707). *bz* = 16: 98.000% (sd. ± 2.244). *bz* = 32: 98.255% (sd. ± 1.165). *bz* = 64: 97.745% (sd. ± 1.204). *bz* = 128: 98.426% (sd. ± 0.809). *bz* = 256: 96.894% (sd. ± 1.593). See Table 3 and Figure 8C. The third LSTM model was a CNN_LSTM, tested with a convolutional layer with five alternatives for the filter-parameter (32, 64, 128, 256, 512), with an output of correct estimations of: *fp* = 32: 96.638% (sd. ± 1.749). *fp* = 64: 97.532% (sd. ± 1.154). *fp* = 128: 98.255% (sd. ± 1.830). *fp* = 256: 99.404% (sd. ± 0.434). *fp* = 512: 99.234% (sd. ± 1.106). See Figure 8.

**Table 2:** Traditional machine learning techniques.

**Model**	**Accuracy (%)**
Gaussian Process classifier	97.447
Extra Trees classifier	95.319
Random Forest classifier	94.894
Gradient Boosting classifier	93.191
Bagging classifier	93.191
K-Nearest-Neighbors classifier	90.638
Support Vector Machine classifier	86.809
Decision Tree classifier	71.915
Naive Bayes classifier	61.277

*Machine learning models taken from Scikit-learn. All models were utilized applying standard parameters explained in the Scikit-learn web tutorials called “Classifier comparison”*.

**Table 3:** Deep learning techniques.

**Models**	**Parameters**	**Accuracy (%)**	**Standard deviation**
CNN	(Filter = 64, Kernel = 9)	96.979	(±0.922)
CNN (Standardization)	(Filter = 64, Kernel = 9)	97.149	(±0.809)
CNN	(Filter = 16)	93.319	(±1.713)
	(Filter = 32)	95.447	(±2.059)
	(Filter = 64)	96.894	(±1.077)
	(Filter = 128)	97.234	(±0.956)
	(Filter = 256)	97.617	(±0.993)
CNN	(Kernel = 2)	97.319	(±0.603)
	(Kernel = 3)	97.702	(±0.545)
	(Kernel = 5)	98.170	(±0.383)
	(Kernel = 7)	97.830	(±0.817)
	(Kernel = 9)	96.723	(±2.738)
3DMultiHeaded_CNN	(*Filter*_1_ = 3, *Filter*_2_ = 5, *Filter*_3_ = 9)	98.553	(±0.340)
Conv_LSTM	(Filter = 64, Batches = 8)	99.234	(±0.707)
	(Filter = 64, Batches = 16)	98.000	(±2.244)
	(Filter = 64, Batches = 32)	98.255	(±1.165)
	(Filter = 64, Batches = 64)	97.745	(±1.204)
	(Filter = 64, Batches = 128)	98.426	(±0.809)
	(Filter = 64, Batches = 256)	96.894	(±1.593)
CNN_LSTM	(Filter = 32)	96.638	(±1.749)
	(Filter = 64)	97.532	(±1.154)
	(Filter = 128)	98.255	(±1.830)
	(Filter = 256)	99.404	(±0.434)
	(Filter = 512)	99.234	(±1.106)

*The report of all models tested. CNN_LSTM reported the best accuracy percentage in the case of the filter parameter setup in 256. Conv_LSTM with a setup of Filter = 64 and Batches = 8 had a high accuracy percentage as well*.

### 4.1. Data Size

**Figure 9:**
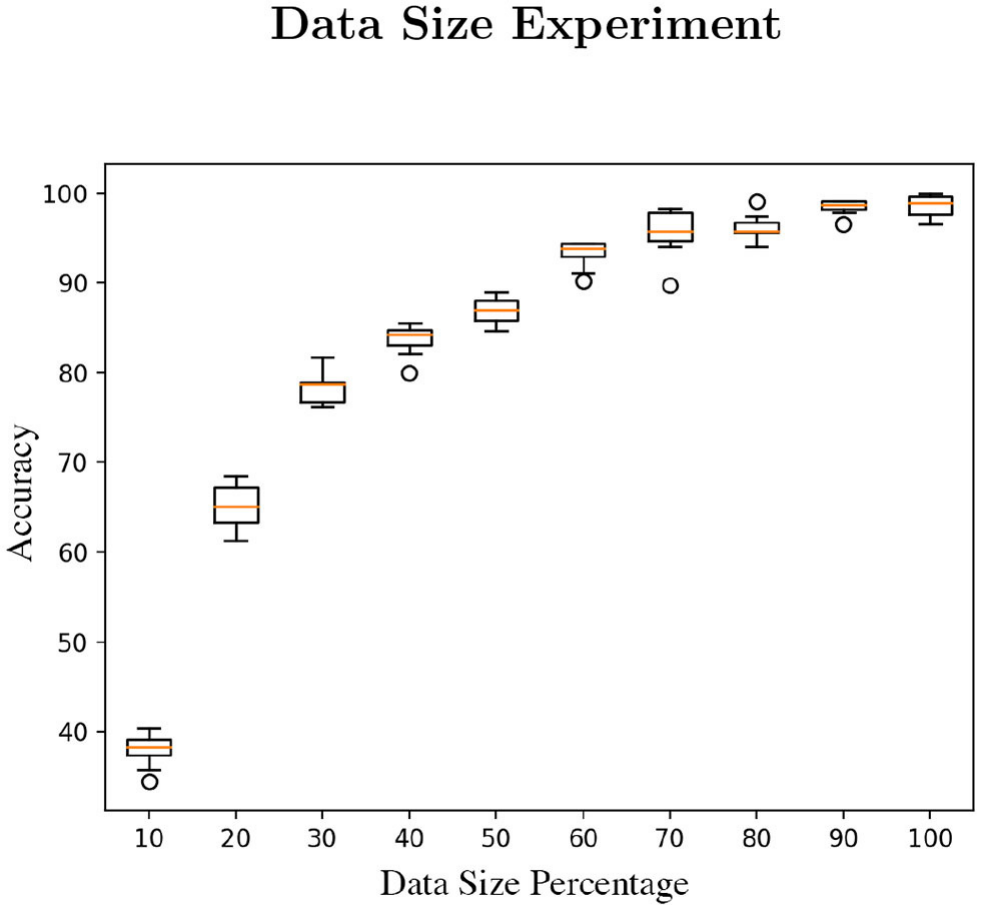
The figure is composed of 10 experiment runs per parameter (percentage of the data used in this study) with 20 epochs each test run.

We have run a computational experiment to determine the minimum size of data applicable to train the RNN models (~28.4 MB). Through this test, we identified the minimum limit in terms of data size to resolve the classification a task. The experiment consists of training using only 10% of the available data, sequentially increasing the size by 10% with each test. We established 20 epochs as the minimum optimal training setup and we have also cleared the session at each test cycle [*tf.keras.backend.clear_session()*]. The result is plotted in Figure 9.

## 5. Discussion

We applied deep convolutional neural network architectures for learning spatiotemporal features in violin bow-stroke gesture recognition. After testing different state-of-the-art models, the Vanilla LSTM was not as accurate (86.383% sd. ± 5.516) as the standard CNN model (97.149 sd. ± 0.809). However, the hybrid model CNN_LSTM showed better results. The architecture of two convolutional layers, with a filter of 512 and a kernel of 3, extracted the features from the time-series sequence observations encoding the global temporal information and the local spatial characteristic of each gesture. The tensors of the CNN layers are projected to the LSTM, which maintains the key features over the different cycles, improving its recognition scores to 99.235% (sd. ± 1.106). In Table 3 the filter = 256 showed high accuracy; however, with a more compact standard deviation than the previous version, it might be the better model for stability.

The traditional ML models resolved the classification problem with good results for the Gaussian Process classifier, with an accuracy report of 97.447%, as well as the Extra Trees classifier, which reported accuracy of 95.319%. Those results confirm that many classifications challenges can be resolved with standard models, taking into account that any particular adaptation or fine-tuning was applied to the ML models, which could also enhance their correct classification percentages.

As shown in Table 4, only the *trémolo* articulation had a precision lower than 90% accuracy. Among the techniques studied, *trémolo* is arguably the least-defined gesture in this context; it can be executed with an arbitrary temporal pattern. However, the architecture identified the gestures, even with different spatiotemporal shapes among the same class, reporting a precision of 100% in almost every trial.

**Table 4:** CNN_LSTM classification report.

**Class**	**Precision**	**Recall**	**F1-score**	**Support**
Martelé	1.00	0.95	0.97	40
Staccato	1.00	1.00	1.00	25
Detaché	1.00	0.96	0.98	25
Ricochet	1.00	1.00	1.00	40
Legato	1.00	1.00	1.0	35
Trémolo	0.89	1.00	0.94	25
Collé	0.96	1.00	0.98	25
Col legno	1.00	0.95	0.97	20
Micro avg	0.98	0.98	0.98	235
Macro avg	0.98	0.98	0.98	235
Weighted avg	0.98	0.98	0.98	235

*The classification report gives a percentage of correct classifications per gesture at a scale of 1:100*.

Related to the data size, after doing the data reduction experiment to establish the minimum requirement to perform the gesture recognition tests, we have clarified that the data published in this study is sufficient to train the RNN models. Based on the results of the experiment (see Figure 9), using about the 60 or 70% of the data, the accuracy reports are already acceptable.

Further work will be needed to develop the gesture-recognition models described here into a feedback system that could be used by musicians in their practice. This system would ideally be co-created with students and teachers to ensure that the feedback is clear and relevant, and the system as easy as possible to operate and understand. Key to this application would be the visualization of performance movements usually hidden to the performer; the graphics presented in Figure 2 and, in particular, Supplementary Figure 4 indicate how a musician might better understand the quality of their gestures in terms of consistency and efficiency. This work would include controlled experimental trials to determine the efficacy of the systems in increasing practice efficiency and, perhaps, reducing physical load and long-term injury resulting from the repetitive motions involved in music performance. The gesture recognition presented is also applicable to other instruments within and beyond the string family, each of which requires a wide set of techniques to master. A further challenge would be to test the same gestures by extracting only the audio features and applying CNN to the resultant audio spectrograms. This approach would provide a significant increase in usability in that musicians would no longer need to purchase, set up, and wear separate IMU sensors and it would remove a significant potential point of failure in a future at-home system for music practice. While these technologies are at present in their early stages, they offer promising potential for a paradigm shift in how musical expertise is developed and shared.

## Data Availability Statement

The raw data supporting the conclusions of this article will be made available by the authors, without undue reservation.

## Author Contributions

DD recorded, processed, and analyzed the motion and audio data, implemented the ML&DNN architectures, and wrote the paper. GW contributed to the music pedagogical framework, collection of performance data, and writing of the paper. RR supervised the methodology and analysis of the data, and contributed to the writing of the paper. All authors contributed to the article and approved the submitted version.

## Conflict of Interest

The authors declare that the research was conducted in the absence of any commercial or financial relationships that could be construed as a potential conflict of interest.

## Footnotes

^1^https://github.com/Dazzid/Applying_Deep_Learning_Techniques_to_Estimate_Patterns_of_Musical_Gesture

^2^http://creativecommons.org/licenses/by-nc-sa/4.0/

^3^https://eater.net/quaternions

^4^https://github.com/Dazzid/Applying_Deep_Learning_Techniques_to_Estimate_Patterns_of_Musical_Gesture
