# Supplementary material for: Applying Deep Learning Techniques to Estimate Patterns of Musical Gesture
Source: Front Psychol. 2021 Jan 5;11:575971. doi: 10.3389/fpsyg.2020.575971 (PMC7813937; doi:10.3389/fpsyg.2020.575971)
Supplement: Supplementary file 1 [file Data_Sheet_1.PDF]

Supplementary Material

1 SUPPLEMENTARY TABLES AND FIGURES

1.1 Figures

The supplementary material consists of three histograms, Euler Angle, Accelerometer and Gyroscope plots of the normalised data under -1 to 1. range. The data is represented by x, y, z axes in light-blue, dark-blue and orange respectively. The fourth plot is the complete exercises per gesture given by the Euler angles from an expert performer.

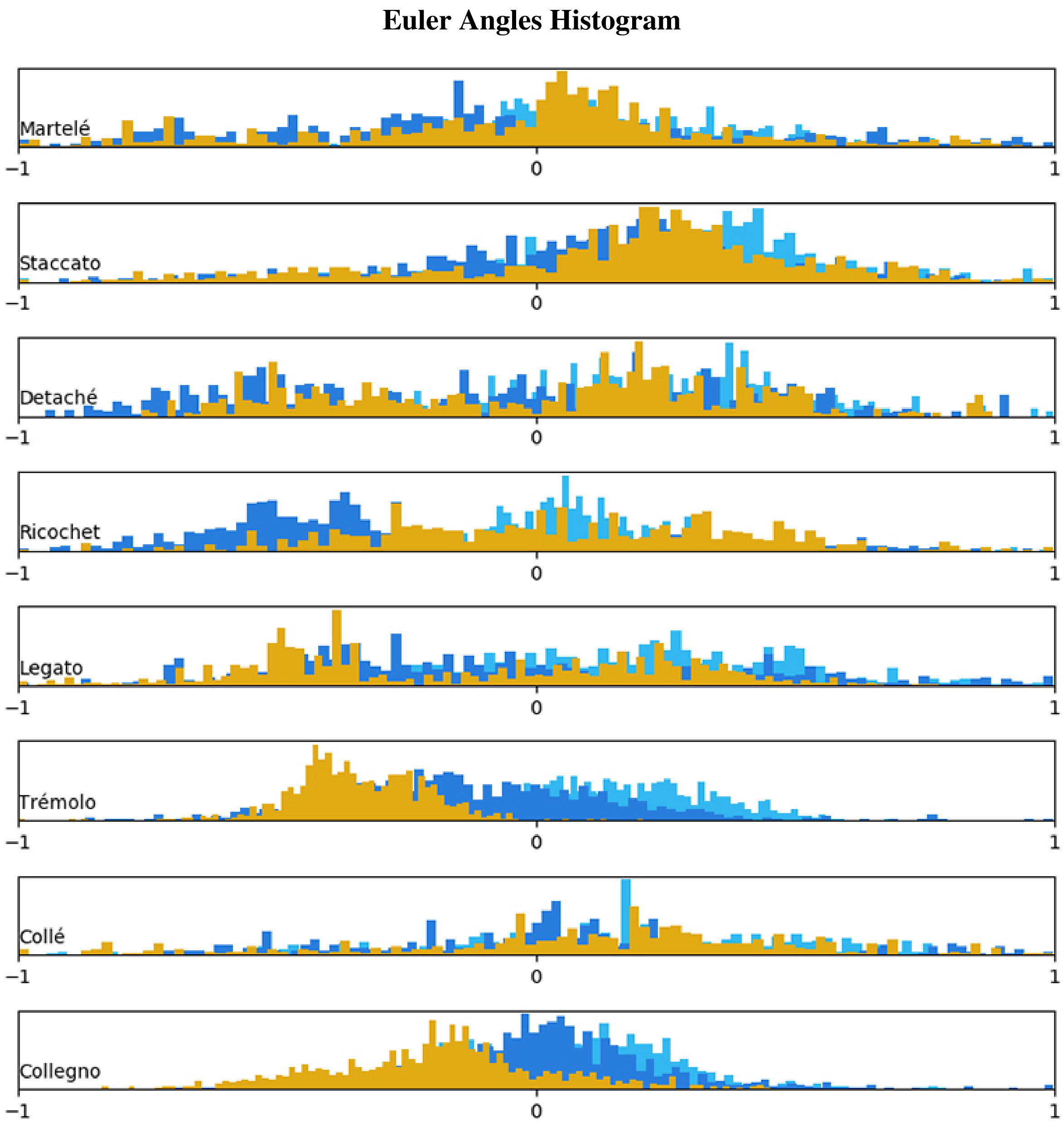

Figure S1. The histogram is the distribution of the data of one subject where the x-axis is shown in light-blue, the y-axis in dark-blue and the z-axis in orange. Euler angles are extracted from the Quaternion data; process explained in Methods Section

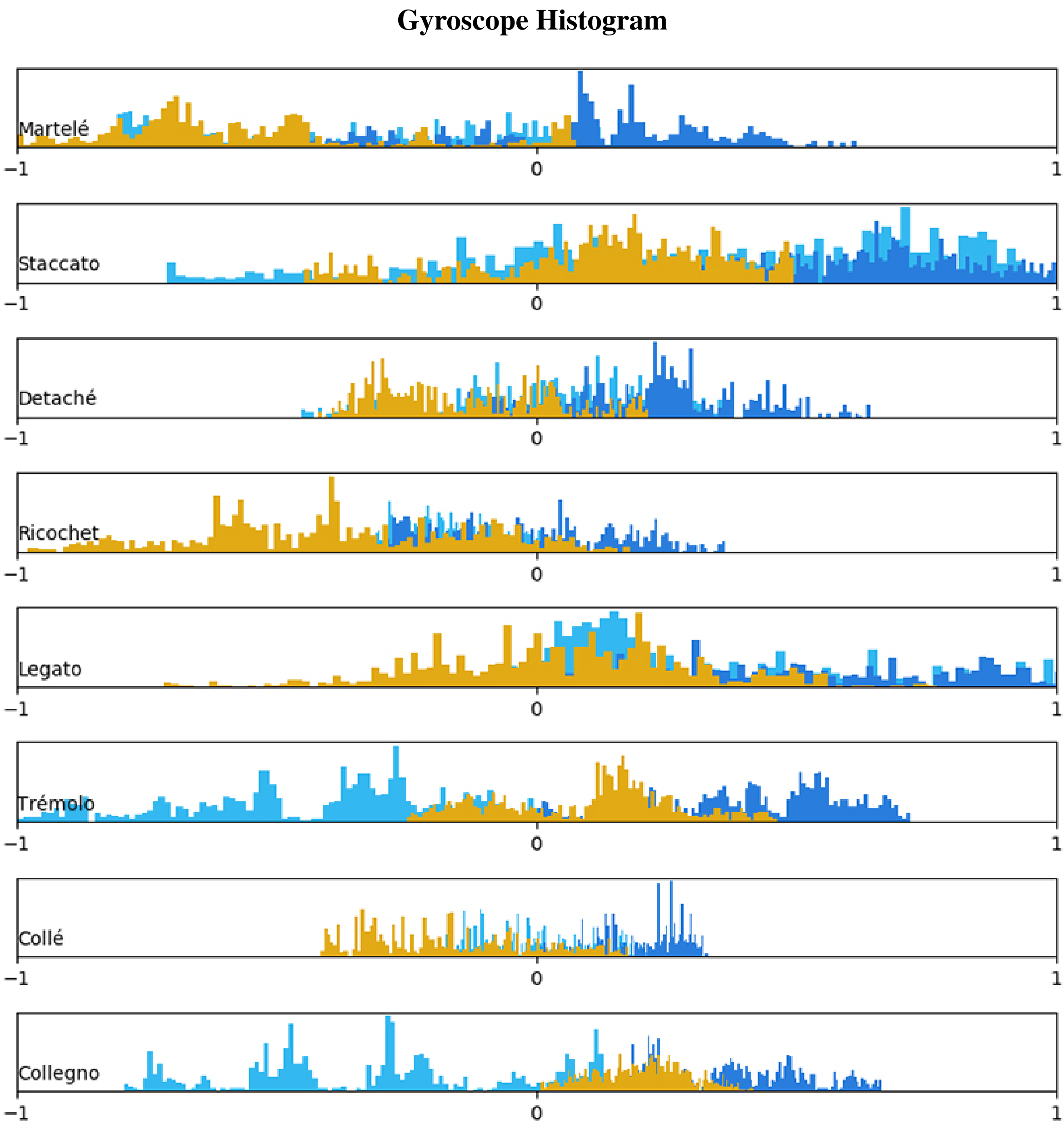

Figure S2. The histogram is the distribution of the data of one subject where the x-axis is shown in light-blue, the y-axis in dark-blue and the z-axis in orange.

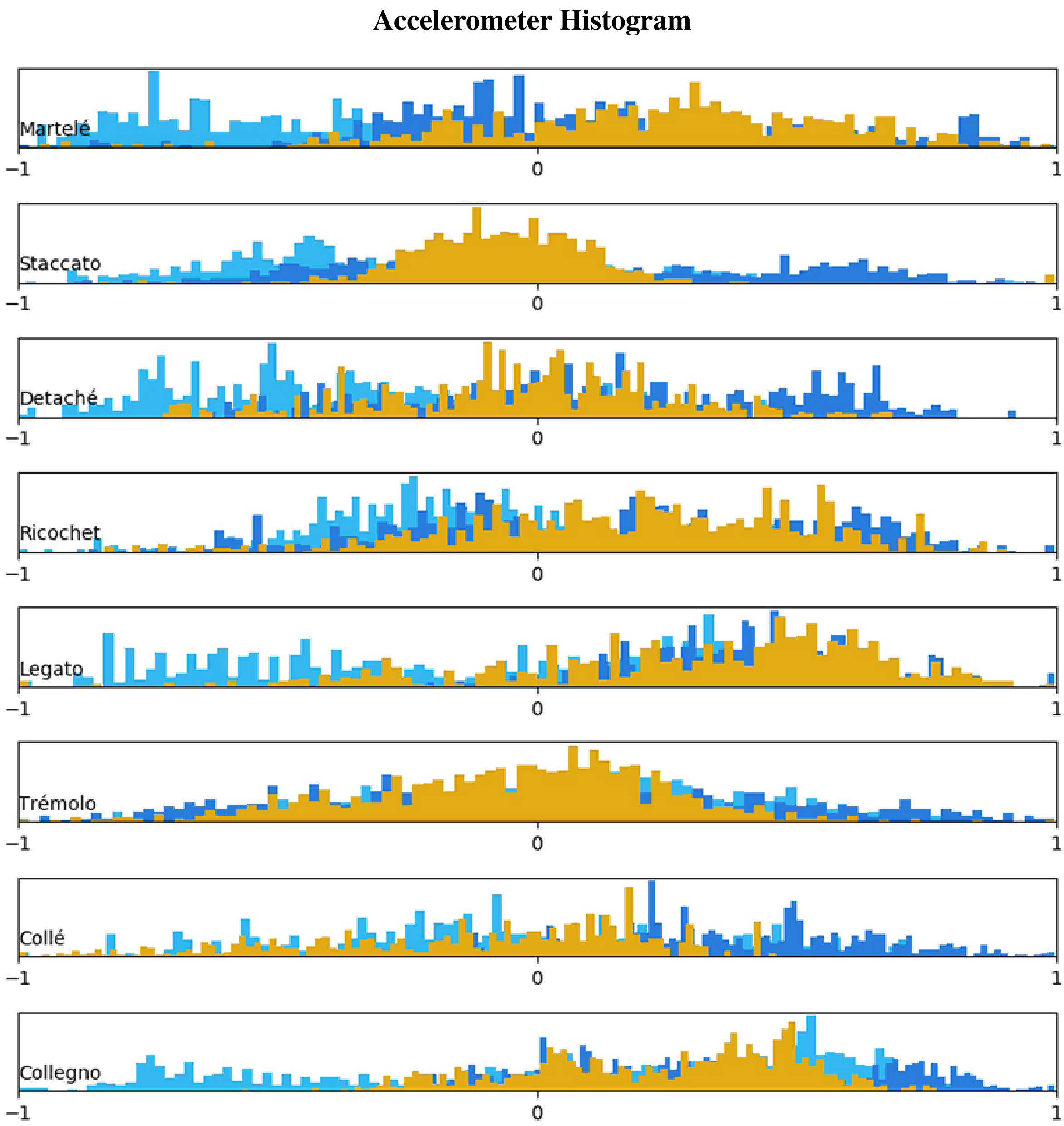

Figure S3. The histogram is the distribution of the data of one subject where the x-axis is shown in light-blue, the y-axis in dark-blue and the z-axis in orange.

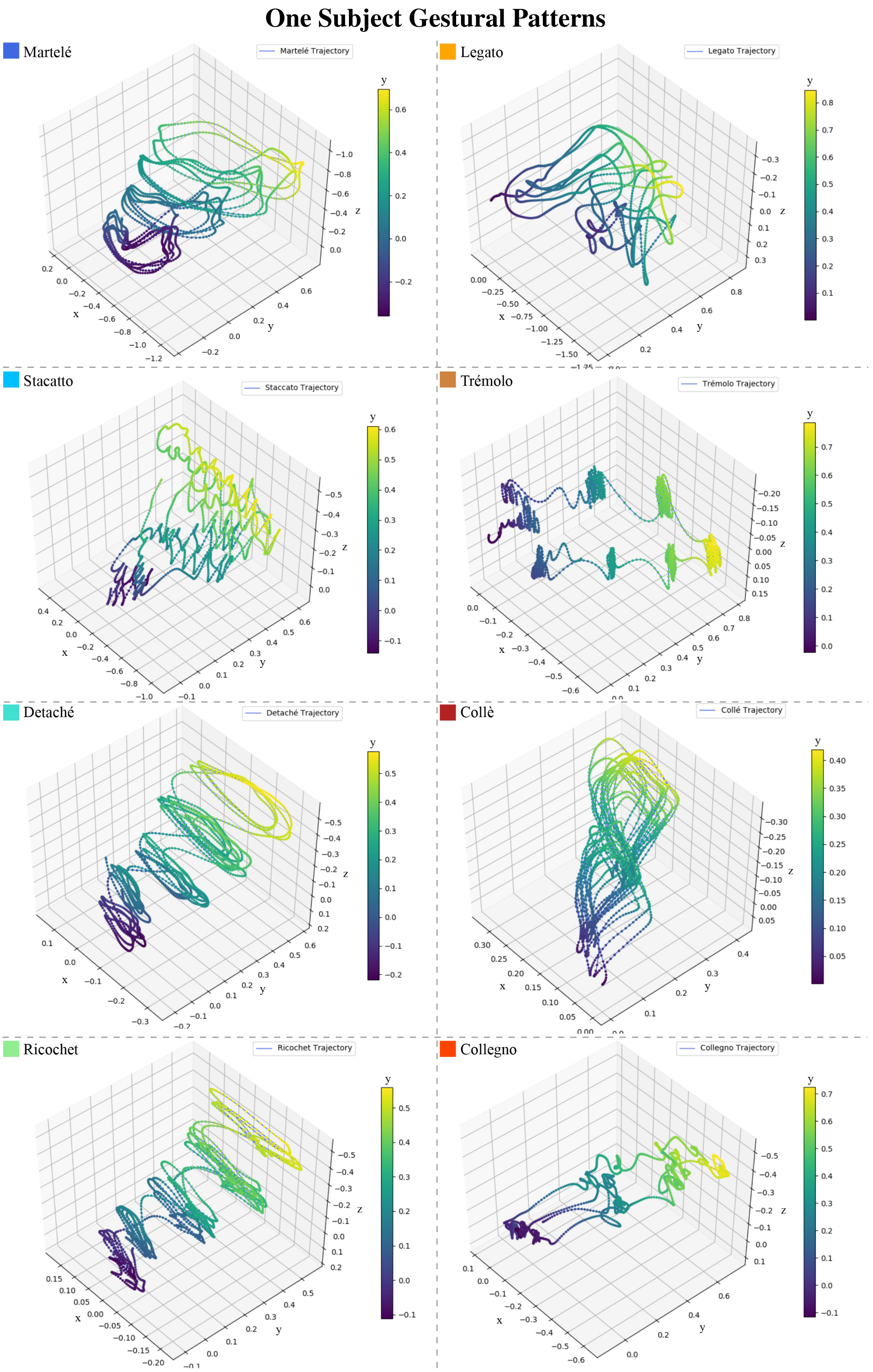

Figure S4. The diagram shows the trajectories of the Myo-Sensor per gesture. Those trajectories are given by the Euler angles taken from one expert performer. The plot provides an insight into how each gesture can be understood as a temporal-signature with specific shapes and times.
